# Utilidad de la Resonancia Magnética Cardíaca con Estrés Farmacológico para valorar Isquemia Miocárdica

**DOI:** 10.47487/apcyccv.v1i1.8

**Published:** 2020-03-30

**Authors:** Sara Ramírez, Esmeralda Paucca

**Affiliations:** 1 Hospital Central de la Fuerza Aérea del Perú - Sección de Cardiología. Lima, Perú. Hospital Central de la Fuerza Aérea del Perú Sección de Cardiología Lima Perú; 2 Instituto Nacional Cardiovascular INCOR- Servicio de Cardiología No Invasiva. Lima, Perú. Instituto Nacional Cardiovascular INCOR Servicio de Cardiología No Invasiva Lima Perú; 3 Clínica Internacional - Unidad de Diagnóstico por Imagen Cardiovascular. Lima, Perú. Clínica Internacional Unidad de Diagnóstico por Imagen Cardiovascular Lima Perú

**Keywords:** resonancia magnética cardíaca, estrés farmacológico, perfusión miocárdica, viabilidad, adenosina, cardiac magnetic resonance, pharmacologic stress, myocardial perfusion, viability, adenosine

## Abstract

La enfermedad cardiovascular es la causa principal de mortalidad y morbilidad en el mundo y se estima que su prevalencia irá en aumento debido al incremento en la prevalencia de los factores de riesgo según la Organización Mundial de la Salud (OMS). En el Perú, la enfermedad cardiovascular es la segunda causa de muerte, por ello la detección de isquemia es una parte importante de la estrategia diagnóstica en pacientes que tienen sospecha de enfermedad coronaria, puesto que es un fuerte predictor de eventos adversos como infarto de miocardio y muerte de origen cardiovascular.

La resonancia magnética cardíaca con estrés farmacológico para evaluar isquemia es una herramienta diagnóstica no invasiva que ofrece muchas ventajas sobre otras técnicas diagnósticas ya que tiene una alta resolución espacial y ausencia de exposición a la radiación. Su precisión diagnóstica para detectar enfermedad coronaria es alta, así como su valor pronóstico en los pacientes con sospecha de esta enfermedad.

En esta revisión se describirá cómo se realiza el estudio de resonancia magnética cardiaca con estrés farmacológico para detección de isquemia y también se discutirá el valor pronóstico y diagnóstico en pacientes con sospecha de enfermedad coronaria.

## Abstract (en)

Cardiovascular disease (CVD) represents the main cause of death in the world. According to the World Health Organization (WHO), its prevalence is estimated to increase considering the high prevalence of cardiovascular risk factors. In Peru, CVD remains the second cause of death in general population. Ischemia represents a strong predictor of future adverse events such as myocardial infarction and cardiovascular death. Accurate detection of ischemia exerts a pivotal role in the diagnostic strategy of patients with suspected coronary disease.

Stress magnetic resonance is a non-invasive diagnostic tool, that offers advantage over other diagnostic techniques, considering that possesses high spatial resolution and no radiation exposure. Its diagnostic precision for detecting coronary artery disease (CAD) is high, in addition of its prognostic value in patients with suspicion of CAD.

In this review we will describe how cardiac magnetic resonance with pharmacologic stress is performed in order to detect ischemia. We will also discuss the diagnostic and prognostic values of this tool in patients with suspicion of CAD.

La enfermedad cardiovascular es la causa principal de muerte en el mundo, con casi 17.6 millones de muertes por año en el 2016, cifra que se espera se incremente a más de 23.6 millones en el año 2030. Esta mortalidad irá en aumento debido al incremento en la prevalencia de los factores de riesgo según la Organización Mundial de la Salud (OMS).^1^ En el Perú es la segunda causa de muerte luego de las enfermedades infecciosas.^2^

La falta o disminución del flujo sanguíneo y por ende de la oxigenación al músculo cardiaco, dan como resultado la progresión de una serie de eventos, que se inician con la aparición de isquemia sub-endocárdica y si el aporte de oxígeno al tejido no mejora, la isquemia se vuelve transmural, seguida por disfunción diastólica, disfunción sistólica, cambios electrocardiográficos y finalmente angina.^3^ La aparición temprana de trastornos de perfusión en esta cascada de eventos (cascada isquémica), nos muestra la importancia de los estudios de perfusión miocárdica para detectar la isquemia desde las etapas más tempranas. Dentro de la cascada isquémica, la primera alteración que se puede evaluar con las técnicas de imagen es el defecto de perfusión y hay varias técnicas de imagen para su identificación. Dentro de ellas están: la perfusión miocárdica por SPECT, la tomografía por emisión de positrones (PET) y la resonancia magnética cardiaca con estrés farmacológico.^4^

Como se ha demostrado en numerosos estudios, al ser comparada con otras técnicas de imagen que también valoran isquemia y teniendo al flujo de reserva fraccional (FFR) como gold standard para valorarlo, la resonancia magnética cardiaca con estrés farmacológico tiene alta precisión diagnóstica comparable con la PET y la perfusión por tomografía, teniendo la ventaja de no usar radiación ionizante.^5^ Para la realización de este tipo de estudio diagnóstico se usan fármacos vasodilatadores: adenosina y dipiridamol, así como dobutamina a dosis altas y el regadenosón. De igual manera también se puede hacer uso de la adenosina trifosfato (ATP) que es un precursor de la adenosina.^6^

## Generalidades del Estudio de RMN con Estrés Farmacológico

El estudio de estrés farmacológico por resonancia magnética cardíaca (RMC), tiene como objetivo identificar la presencia de estenosis coronarias que generen compromiso hemodinámico significativo. La identificación de isquemia se realiza mediante un agente vasodilatador o inductor de hiperemia (como la adenosina o el dipiridamol) aplicando secuencias de perfusión de primer paso, o bien mediante un inotrópico positivo (dobutamina) cuyo efecto se evalúa por medio de las secuencias de cine en eje corto para análisis de la función contráctil. Este último fármaco es una opción en aquellos pacientes en los que no se puede utilizar la adenosina, o bien en aquellos pacientes con origen anómalo de las coronarias o puentes intramiocárdicos en el que la respuesta a un estímulo inotrópico potente es preferible para identificar la repercusión funcional de la patología subyacente.^7^

**Figura 1:**
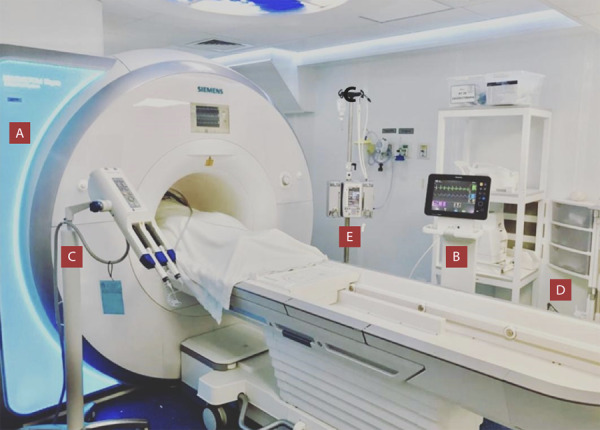
A. Resonador Siemens MAGNETOM Skyra 3T Clínica Internacional. B. Monitor cardíaco compatible con campo magnético. C. Inyector de medio de contraste. D. Coche de paro. E. Bomba de infusión compatible con campo magnético.

Se requiere un monitor cardíaco compatible con el campo magnético de la sala de resonancia para valorar la presión arterial, frecuencia cardíaca y saturación de oxígeno, así como una bomba infusora. Además se debe tener al alcance un coche de paro para realizar maniobras de reanimación cardiopulmonar avanzada.^8^ (Figura 1)

Está contraindicado el uso de adenosina en pacientes con EPOC, bloqueo auriculoventricular de segundo o tercer grado, bradicardia sinusal (menor de 45 latidos por minuto), hipotensión arterial sistémica (sistólica menor de 90 mmHg). En el caso de la dobutamina, esta se contraindica en pacientes con hipertensión arterial severa (>220/120 mmHg), angina inestable, estenosis aórtica severa, arritmias cardíacas complejas, miocardiopatía hipertrófica obstructiva, miocarditis, pericarditis y endocarditis activas, en pacientes con infarto agudo de miocardio está contraindicado dentro de los primeros 15 días.^9^

En la preparación del paciente, se indica la suspensión de cafeína, teofilina y derivados (café, té, medicaciones con cafeína) en caso de usar adenosina y dipiridamol. Se ha estudiado que la ingesta de café antes de las cuatro horas previas al estudio de resonancia magnética cardiaca con adenosina disminuye la reactividad de manera significativa al fármaco estresor, por lo que es importante orientar al paciente de manera adecuada previamente a la realización de este tipo de estudios.^10^ En el caso de usar dobutamina, se debe suspender los betabloqueadores debido a que estos actúan de manera directa y competitiva sobre los receptores beta 1 y actuarían como antagonistas de los efectos del fármaco. De igual manera se debe suspender los nitratos ya que disminuyen la sensibilidad de la respuesta al estrés con dobutamina.^8^

Es muy importante además conocer los posibles efectos adversos, los cuales deben ser explicados al paciente para su colaboración durante la adquisición de las imágenes, en el caso de la adenosina ésta puede causar dolor precordial, palpitaciones, sensación de falta de aire y rubor. Los efectos adversos mayores incluyen bloqueo auriculoventricular transitorio, hipotensión, taquicardia sinusal y broncoespasmo severo. Por otro lado, la dobutamina puede ocasionar dolor precordial, palpitaciones o reacción hipertensiva severa; asimismo (aunque menos frecuente), se puede producir fibrilación ventricular, taquicardia ventricular sostenida y, en casos raros, infarto agudo de miocardio.^11^

El equipo de trabajo (médico cardiólogo y/o radiólogo, así como el tecnólogo médico), debe tener un entrenamiento adecuado en la adquisición, interpretación y manejo de posibles complicaciones durante el estudio.^8^

## Agentes Estresores y Medios de Contraste

Dentro de los fármacos comúnmente usados para el estrés farmacológico en RMC están la adenosina y el dipiridamol (tiempo de vida media de 30 minutos luego de la inyección), los cuales inducen vasodilatación. En el 2008 la Agencia de Administración de Alimentos y Medicamentos de Estados Unidos (FDA por sus siglas en inglés) aprobó el uso de regadenosón, el cual viene siendo usado con más frecuencia en los últimos años ya que tiene un tiempo de vida media aproximado de 10 segundos.^11^

La adenosina actúa sobre el músculo liso de los vasos sanguíneos condicionando vasodilatación. El dipiridamol inhibe la recaptación celular y el metabolismo de la adenosina, y por lo tanto condiciona un incremento de la concentración de la misma. El regadenosón es un agonista del receptor de la adenosina.^12^ (Tabla 1)

**Tabla 1:** Características de los agentes de estrés

Agente de estrés	Dosis (Velocidad de infusión)	Mecanismo de acción
Dipiridamol	0.56 mg/kg (4 minutos)	Inhibe recaptación de adenosina
Adenosina	140-210 μg/kg/min (3-5 minutos)	Activación de receptores A_1_, A_2A_, A_2B_ y A_3_
Adenosina 5 trifosfato (ATP)	160 μg/kg/min (3-5 minutos)	Activación de receptores A_1_, A_2A_, A_2B_ y A_3_
Regadenoson	0.4 mg (bolo)	Activación del receptor A_2A_

La dobutamina también se usa para la RMC con estrés farmacológico y valoración de perfusión miocárdica. Este fármaco incrementa la frecuencia cardíaca, la presión arterial y la contractibilidad de forma similar al ejercicio. El protocolo se realiza con incrementos progresivos de la dosis del fármaco. En cada etapa se adquieren imágenes del ventrículo izquierdo para valorar los cambios relacionados a la isquemia así como la disfunción sistólica. A dosis bajas, también puede ser utilizada para la evaluación de las anormalidades de la pared, lo cual daría una información adicional a la secuencia de realce tardío cuando se desea valorar viabilidad miocárdica.^13^

El agente estresor que usamos en nuestro país por su disponibilidad, mayor rapidez, mejor tolerabilidad y más amplio margen de seguridad es la adenosina (vida media corta de 10 a 30 segundos, por lo que los efectos adversos pasan rápidamente). Se recomienda tener aminofilina y betabloqueadores endovenosos al alcance para tratar posibles efectos adversos prolongados. Este agente estresor tiene la propiedad de inducir vasodilatación coronaria máxima a través de la activación de los receptores de purina en la membrana celular (A1 y A2). Clínicamente la vasodilatación coronaria puede ser valorada con el incremento de la frecuencia cardíaca > 10 lpm y/o caída de la presión arterial sistólica > 10mmHg.^14^

El gadolinio es el contraste standard utilizado en los estudios de resonancia magnética. Los quelatos del gadolinio son solubles al agua y por ellos se difunden rápidamente en el espacio extracelular; sin embargo no son capaces de atravesar las membranas intactas de los miocitos. Dentro de las reacciones adversas leves se encuentran nauseas y erupciones dérmicas. Las reacciones adversas serias (incluyendo reacciones anafilácticas) que ponen en riesgo la vida del paciente se encuentran en el rango de 1 en 200,000 a 1 en 400,000. La fibrosis nefrogénica sistémica, la cual es una enfermedad rara irreversible que está relacionada con la administración de gadolinio en pacientes con enfermedad renal crónica en estadio terminal, nos obliga a que dentro del protocolo de preparación del paciente se evalúe la función renal previa a la administración del fármaco con la finalidad de evitar la ocurrencia de esta enfermedad.^15^

Las contraindicaciones para el uso de los fármacos descritos y del medio de contraste, así como quienes no pueden realizarse el estudio se describen en la Tabla 2.

**Tabla 2:** Contraindicaciones para varios componentes en resonancia magnética cardíaca

Agente	Contraindicación
Adenosina	Asma Enfermedad pulmonar obstructiva crónica Bloqueo auriculoventricular de II o III grado Frecuencia cardíaca < 45 lpm
Dobutamina	Angina inestable o infarto de miocardio reciente (menos de 1 semana) Estenosis aórtica severa Cardiomiopatía hipertrófica obstructiva Historia reciente de arritmia potencialmente mortal Hipertensión no controlada
Gadolinio	Disfunción renal severa (TFG < 30 ml/min/1.73 m^2^)
Resonador	Claustrofobia Objetos metálicos no compatibles con resonancia magnética (incluyendo estimuladores nerviosos o implante coclear)

TFG: Tasa de filtración glomerular

## Protocolo de Adquisición

El déficit de perfusión se detecta mucho más fácilmente bajo condiciones de estrés, que en RMC se logra bajo estrés farmacológico. Los vasos coronarios con estenosis significativas son incapaces de responder a la estimulación vasodilatadora del fármaco (adenosina) de igual manera que los vasos normales, condicionando una diferencia en el flujo miocárdico.^16^

En la secuencia de perfusión miocárdica por RMC, el contraste ingresa rápidamente y se difunde desde el espacio vascular al espacio extracelular, así que la perfusión debe ser valorada durante el primer paso del contraste dentro del miocardio. Por eso se utiliza también el término “perfusión de primer paso”. La secuencia de perfusión del primer paso es una secuencia saturation recovery potenciada en T1, en 3 planos en eje corto (basal, medial y apical), siendo adquirida primero la secuencia con estrés farmacológico y luego de 10 minutos de concluida se adquiere la secuencia de reposo.^17^ El protocolo indica que se debe adquirir la secuencia de perfusión de primer paso bajo infusión de adenosina a 140 ug/kg/min durante los 4 primeros minutos. Si no se logra el efecto vasodilatador (incremento de la frecuencia cardiaca al menos 10 lpm y/o disminución de la presión arterial sistólica al menos 10mmHg), se aumenta la infusión a 170 ug/kg/min durante 2 minutos más.^8^

Recientemente se ha propuesto un nuevo signo como marcador de respuesta adecuada a los fármacos vasodilatadores usados en los estudios de RMC con estrés farmacológico. Se trata de una perfusión reducida del bazo durante la infusión del fármaco estresor (el bazo se muestra hipointenso cuando hay respuesta estresora adecuada). Las bases fisiológicas para este fenómeno se deben a que el volumen de sangre se reduce significativamente durante el ejercicio debido a la redistribución del flujo esplácnico, y puede objetivarse como una hipointensidad en las secuencias de perfusión en resonancia magnética. El grado de reducción del volumen esplénico es proporcional a la sobrecarga de trabajo, es independiente del gasto cardiaco y está relacionado a la vasoconstricción mediada por adenosina.^18^

**Figura 2:**
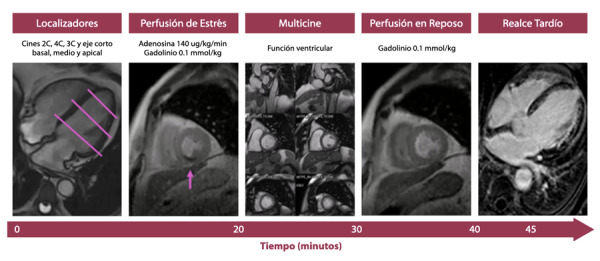
Protocolo de resonancia magnética de estrés con adenosina

Además, debe tenerse en consideración que como parte de la evaluación en el protocolo de estrés, se adquieren las secuencias de función ventricular (cines en 2C, 3C y 4C), así como las secuencia de multicine (que se refiere a múltiples cortes en eje corto que abarcan ambos ventrículos de base a ápex ) y las secuencias de realce tardío para valoración de viabilidad (2C, 3C, 4C y eje corto), con el mismo número de cortes realizados para la función ventricular.^19^ (Figura 2)

## Post-proceso de Imágenes

### Valoración de la función ventricular izquierda y derecha

Se realiza un análisis cualitativo que incluye la evaluación dinámica de la motilidad global y segmentaria del ventrículo izquierdo (VI) y un análisis cuantitativo que incluye el cálculo de volumen telediastólico y telesistólico de los ventrículos, fracción de eyección de ambos ventrículos, volumen sistólico, gasto cardíaco, masa del VI y valores indexados al área de superficie corporal de todos, excepto de la fracción de eyección. Si no hay evidencia de shunts intra o extracardíacos, el volumen sistólico de ambos ventrículos debe ser casi igual^.(^^20^

### Valoración de la perfusión miocárdica

La perfusión coronaria es el factor primario que altera la concentración de gadolinio en el tejido miocárdico. El análisis se realiza con la finalidad de identificar los segmentos con hipoperfusión miocárdica, comparando las imágenes en reposo y estrés para identificar defectos inducibles de perfusión y artefactos. Se observa que los segmentos que están irrigados por una arteria coronaria con estenosis significativa (la cual condiciona compromiso hemodinámico), mostrará una hipointensidad subendocárdica o transmural lo que indicará el territorio con isquemia.^8^

Se considera un defecto de perfusión inducible cuando el contraste llega al miocardio del VI, persiste después de alcanzar la mayor intensidad en el miocardio (por más de 4 latidos usualmente), es mayor de un píxel de ancho, mas prominente en la porción sub-endocárdica, el defecto retorna hacia el subendocardio, está presente en el estrés pero no en el reposo o con menor intensidad en el reposo y sigue el territorio de una o más arterias coronarias.^20^ (Tabla 3)

**Tabla 3:** Análisis visual de imágenes de perfusión cardíaca

Reposo	Realce tardío	Interpretación
Negativo	Negativo	Normal
Negativo	Positivo	Infarto
Negativo	Negativo	Isquemia
Positivo	Positivo	Infarto
Positivo	Negativo	Artefacto Isquemia en reposo Llegada tardía de contraste

**Figura 3:**
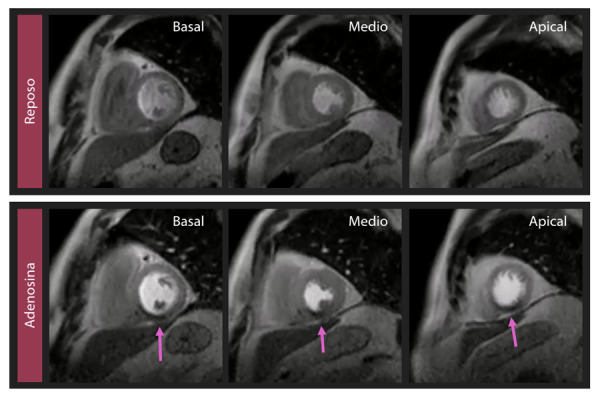
Se observa defecto de perfusión inducible con adenosina (que no se observa en la perfusión en reposo) a nivel inferior basal, medial y apical, correspondiente a 3/16 segmentos. Se considera isquemia significativa el compromiso > 2/16 segmentos.

Se debe informar la localización y extensión de el o los defectos inducibles, utilizando el modelo de la segmentación según Cerqueira de 16 segmentos, mencionar la cantidad de segmentos involucrados (isquemia significativa > 2/16 segmentos) y describir la transmuralidad de un defecto de perfusión. Cabe mencionar que en la enfermedad microvascular se observa una reducción concéntrica de la perfusión (defecto en anillo subendocárdico).^21^ (Figura 3) Es también importante diferenciar el miocardio perfundido a nivel subendocárdico de artefactos de borde oscuro que son comunes de presentarse a nivel de la interface sangre-miocardio como el anillo de Gibss (fenómeno de Gibbs).^20^ Las diferencias en la intensidad regional pueden ser causadas por una pérdida de la perfusión miocárdica distal y una re-dirección del flujo a las capas epicárdicas. La vasodilatación sistémica puede también alterar el flujo colateral de alta resistencia.

### Valoración de la viabilidad con la secuencia de realce tardío con gadolinio

En este punto se analiza la retención miocárdica de gadolinio, la cual, cuando es secundaria a enfermedad coronaria, compromete siempre el sub-endocardio y puede llegar a ser transmural, además de seguir un territorio arterial coronario. Se informa la extensión usando el modelo de 17 segmentos de la American Heart Association (AHA) para valorar viabilidad, estimando siempre el promedio de extensión transmural del realce tardío en cada segmento (0%, 1-25%, 26-50%, 51-75% y 76-100%), considerando que los segmentos con retención de gadolinio mayor de 75% no son viables y los menores de 25% son viables. En los segmentos que están entre 25 y 75% se puede valorar además la viabilidad mediante infusión de dobutamina a dosis bajas.^8^^,^^13^^,^^20^

## Evidencia de la Precisión Diagnóstica de la RMC y su Comparación con otras Técnicas de Imagen

La RMC ha demostrado ser una herramienta útil para la estratificación del riesgo en pacientes con síntomas de isquemia.^22^ Sabiendo que la enfermedad coronaria es la mayor causa de morbilidad y mortalidad en pacientes con diabetes mellitus, un estudio el 2011 en pacientes diabéticos para evaluar la sospecha de isquemia miocárdica, evidenció un incremento de eventos cardíacos mayores de hasta tres veces en aquellos en los que se demostró isquemia por perfusión miocárdica realizada por RMC.^23^

La angiografía coronaria invasiva es el “gold standard” en la evaluación de la severidad de las obstrucciones coronarias. Sin embargo, se debe tener en cuenta que es un procedimiento invasivo, costoso y con uso de radiación ionizante, por lo que no es un método diagnóstico con fines de screening en pacientes con sospecha de enfermedad coronaria.^24^ Un metaanálisis de Nandalur et al, demostró que la RMC con estrés farmacológico tiene una sensibilidad de 0.91 y una especificidad de 0.81 para la detección de isquemia miocárdica en el contexto de lesiones coronarias obstructivas al ser comparado con la angiografía coronaria.^25^

La RMC con estrés farmacológico para valorar perfusión miocárdica ofrece una excelente resolución espacial con vóxel de 3 mm (comparado con los 10 mm del SPECT y los 6 mm de la PET). Esto nos permite la detección de defectos de perfusión en el subendocardio, que en otras modalidades podría no detectarse.^26^

Un metaanálisis ha revelado una sensibilidad por encima del 89% y una especificidad del 76% para la detección de enfermedad coronaria usando RMC. El estudio CE-MARC ha demostrado mayor sensibilidad (87% versus 67%, p<0.0001) y similar especificidad (83% versus 83%, p= 0.916) para valorar enfermedad coronaria por RMC vs SPECT.^27^^-^^29^

Una de las observaciones realizadas a la precisión diagnóstica de la RMC, era que los primeros estudios utilizaron una técnica diagnóstica anatómica como patrón de referencia, es decir la coronariografía invasiva, mientras que la resonancia magnética cardiaca es una prueba funcional. Así, un estudio reciente muestra la precisión diagnóstica de la RMC frente al flujo de reserva fraccional (FFR) como “gold standard” para valoración de isquemia, mostrándose que esta técnica de imagen no invasiva puede descartar de manera precisa la enfermedad coronaria obstructiva con significancia hemodinámica cuando es comparada al FFR.^26^ Watkins et al. compararon la precisión diagnóstica de la RMC versus el FFR (donde un valor menor de 0.75 indica estenosis significativa), encontrando que la sensibilidad y especificidad del análisis visual de la perfusión por RMC con estrés farmacológico para detectar enfermedad coronaria con estenosis significativa identificada por FFR fue 0.82 y 0.94 respectivamente, con un área bajo la curva (AUC) de 0.92.^30^ Es por ello que la perfusión de estrés con RMC puede detectar estenosis coronaria con significancia funcional dada su excelente precisión diagnóstica. 

Un metaanálisis publicado por Li et al, se valoró la precisión diagnóstica de la RMC por estrés farmacológico versus FFR, donde la sensibilidad, especificidad y área bajo la curva fueron 0.90, 0.87 y 0.95 al analizar por paciente, y 0.89, 0.86 y 0.93 al analizar por vaso.^31^ Otro estudio valoró la precisión diagnóstica de la RMC con estrés farmacológico al igual que otras modalidades que valoran perfusión miocárdica como el PET, SPECT, perfusión por tomografía computarizada y ecocardiografía de estrés farmacológico y se comparó con el FFR como referencia. Se concluyó que la RMC con estrés farmacológico tiene una sensibilidad de 89% y una especificidad de 87% y tiene mejor precisión que el SPECT que tiene una sensibilidad del 74% y una especificidad del 79% o que la ecocardiografía con estrés la cual tiene una sensibilidad de 69% y una especificidad del 84%.^32^^)^ La RMC con estrés farmacológico alcanza alta precisión diagnóstica, la cual es similar a la perfusión realizada por PET que tiene una sensibilidad del 84% y una especificidad del 87% y de la perfusión por tomografía computarizada que tiene una sensibilidad del 88% y una especificidad del 80%.^33^

Pacientes sin evidencia de isquemia por resonancia magnética cardiaca tienen una tasa de eventos anuales menor del 1% para muerte de origen cardiovascular o infarto no fatal, mientras que aquellos pacientes con isquemia en esta prueba tienen una tasa de eventos de 5% aproximadamente al año.^34^

Recientemente se ha comparado el valor pronóstico de la RMC en pacientes con sospecha de enfermedad coronaria en un seguimiento de 5 años a partir del estudio CE-MARC. Los resultados indican que la resonancia magnética cardíaca es un fuerte predictor de eventos cardíacos adversos mayores (MACE) en comparación con el SPECT, independientemente de los factores de riesgo cardiovascular, resultados de la angiografía coronaria o tratamiento del paciente.^35^^,^^36^

La guía de síndromes coronarios crónicos publicada en el 2019 por la European Society of Cardiology (ESC), le da a la resonancia magnética cardíaca con estrés farmacológico una recomendación IB para valoración de isquemia, donde la isquemia significativa está definida por la presencia de defectos reversibles en 2 o más de 16 segmentos valorados por perfusión miocárdica, por resonancia magnética cardíaca con estrés farmacológico (4 de los 32 segmentos si están divididos en subsegmentos endocárdico y epicárdico), o al menos 3 nuevos segmentos disfuncionantes durante la resonancia con estrés farmacológico con dobutamina.^37^^-^^39^

## Conclusiones y Aplicaciones en la Práctica Clínica

La RMC tiene gran potencial pues, en un solo estudio, se puede valorar la función cardíaca bi-ventricular, los defectos de perfusión miocárdica para valorar isquemia y dar información acerca de la viabilidad miocárdica por la secuencia de realce tardío; teniendo en cuenta que es además, la técnica ideal para la valoración de función y volúmenes cardíacos y sin el uso de radiación ionizante. Las contraindicaciones para resonancia magnética cardíaca deben ser identificadas sobre todo para el uso de agentes estresores además del uso de contraste. 

Existe evidencia considerable para el uso de RMC en la valoración de isquemia en pacientes con enfermedad coronaria conocida o con sospecha de la misma.

## Financiación:

Ninguna
